# Disturbed body schema, perceptual body image, and attitudinal body image in patients with borderline personality disorder

**DOI:** 10.3389/fpsyt.2023.1168611

**Published:** 2023-09-26

**Authors:** Dorottya Szily, Rebeka Kelemen, Zita S. Nagy, Dominik Szabó, Zsolt Szabolcs Unoka

**Affiliations:** ^1^Doctoral School of Mental Health Sciences, Semmelweis University, Budapest, Hungary; ^2^Independent Researcher, Budapest, Hungary; ^3^OMINT-National Institute of Medical Rehabilitation, Budapest, Hungary; ^4^Department of Psychiatry and Psychotherapy, Semmelweis University, Budapest, Hungary

**Keywords:** borderline personality disorder, body schema, perceptual body image, attitudinal body image, body portraying method

## Abstract

**Background:**

Borderline personality disorder (BPD) is a severe mental disorder that affects attitudes toward the body. However, whether this condition also affects body schema and perceptual body image remains unclear. Previous questionnaire-based studies found dissatisfaction with one's body in patients with BPD. In addition to attitudinal body image, our study investigates whether body schema and perceptual body image are disturbed in patients with BPD.

**Method:**

Our study included 31 patients diagnosed with BPD (25 women) and 30 healthy individuals (19 women) (M_age_ = 29 for both groups). The SCID-5-PD interview was used to determine personality disorder. Attitudinal body image was measured using the Body Attitude Test (BAT) factors. Body schema and perceptual body image were measured by two conditions of a body representation task, the body portraying method (BPM).

**Results:**

BPD patients achieved higher scores in all three BAT factors and were more susceptible to misinformation in both conditions of BPM. Based on the results, BPD patients appear to have more negative attitudes toward their bodies and worse perceptual body image and body schema.

**Conclusion:**

The novel finding of our study is that, besides the previously found attitudinal dissatisfaction with the body, individuals with BPD also show disturbances at the levels of body schema and perceptual body image. Our findings concerning disturbances in body schema and perceptual body need further research into their etiological factors and provide new therapeutic targets for the treatment of BPD.

## 1. Introduction

### 1.1. Perception of the body in borderline personality disorder

Borderline personality disorder (BPD) is a severe mental disorder that affects 1–2% of the general population and 10–20% of psychiatric patients (1). Characteristics of BPD are emotional lability, dysfunction of emotion regulation, and unstable relationships. Patients with BPD often experience severe traumatic events, such as sexual, physical, or verbal abuse, violence, parental separation, and childhood illnesses. They are also more likely to have a first-degree relative with a psychiatric disease (2). Symptoms frequently impact various cognitive aspects, such as overvalued ideas of being guilty, experiences of dissociation in depersonalization and derealization, quasi-psychotic or psychotic-like symptoms, or occasionally reality-based delusions and hallucinations (1, 3). Not only cognition but perception can be altered: patients with BPD showed enhanced sensitivity to fearful expressions and impaired facial emotion recognition of disgust (4), higher pain endurance and tolerance (5), and felt pleasant touch rougher and less intense than healthy controls (6). Several areas of patients with self-experience of BPD are also disturbed: not only identity (7) but the experience of body ownership can be altered or unstable (8). This concept encompasses feelings and thoughts such as a body part not belonging to one's own body or even feeling as if it has disappeared (8, 9). The degree of body ownership was found to be the lowest in BPD patients with severe current symptoms compared to recovered BPD or healthy individuals (8).

Specific BPD symptoms are also strongly associated with dissatisfaction with one's body. Both identity disruption and identity distress can predict appearance evaluation and body satisfaction (10, 11). Patients with BPD often have negative attitudes, lower self-esteem, and a higher level of discomfort related to their bodies (12). They have high levels of dissatisfaction and distrust in their bodies and consider appearance an essential factor for happiness. Moreover, certain authors found that more than half of BPD patients have comorbid body dysmorphic disorders (13). Finally, it was found that borderline personality disorder symptom scores among women in psychiatric outpatient settings were negatively associated with self-rated bodily attractiveness and facial attractiveness and with higher social avoidance due to body image concerns (14).

The studies mentioned above are based on self-reported ratings of body-related experiences and attitudes. However, there is a gap in the literature, as no study has explored the schema of the body and perceptual body image among people living with BPD. According to the review of Kaufman and Meddaoui (15), although identity disturbance is a core feature of BPD, it is understudied and requires further investigation. Furthermore, body image is strongly linked to identity development (16). Based on these, there is a need for a deeper understanding of the disturbances at different layers of body image. New insights at the level of the schema of the body and perceptual body image may also provide new targets for therapeutic interventions.

### 1.2. Different levels of body representation

The idea that the internal model of our body comes from different sources emerged at the beginning of the 20th century (17, 18). Since then, numerous studies focused on body representation have been published across different scientific fields, including clinical psychology, neuropsychology, neurology, and philosophy.

Recent research from cognitive, developmental, and clinical studies suggests that our experiences and knowledge of our bodies are organized within a complex system where different body representations form an integrated unit (18). According to this perspective, this system is grounded in somatosensory inputs from various channels (e.g., skin, muscles, joints, and other deeper tissues) and verbal-conceptual information (e.g., names of body parts and evaluations of body parts). This system constantly evolves by integrating diverse information types (18–20).

Information from and about the body is processed in the cognitive system at different stages or levels (18). Information could be stored on a fundamental, non-declarative level (body schema) and on a more conscious, declarative level (body image). Gallagher (21) defined body schema as a pre-personal unconscious representation that is strongly related to the body's functions. Body image encompasses multiple representations of the body. The two main components examined in this study are perceptual body image and attitudinal body image (18, 22). Perceptual body image encompasses all conscious perceptual information about the body, including its shape, position, or weight. Attitudinal body image encapsulates our cognitive and emotional attitudes toward our bodies.

### 1.3. Aim of our study

BPD significantly influences the cognitive and affective aspects of the individual's body perception (10). Furthermore, it can be assumed that in this disorder, non-conscious body representations, which are grounded on somatosensory stimuli, may also be affected. Most psychotherapeutic interventions used in treating BPD primarily address body image issues at the explicit level by focusing on thoughts and emotions, with relatively little emphasis on the implicit, procedural-level bodily experiences (23). Considering all of these factors, a comprehensive exploration of various levels of body representation may provide new targets for the treatment of BPD.

Initial studies on body image and BPD primarily focused on attitudes and subjective experiences about the body. The body portraying method (BPM) is a new and promising method to investigate different levels of perception of the body (24). This instrument enables the measurement of multiple aspects of body representation by having participants touch specific body parts, mark their locations, and then reproduce these sensations on paper (similar to a mirror reflection). For a detailed description, see the *Method* section. The BPM measures two aspects of body representations: first, the body schema, and second, the perceptual body image. First, body schema is the internal, non-declarative, multimodal representation of body structure and size. Second, perceptual body image is the visual-symbolic offline declarative representation based on the participants' internal visually coded image of their bodies. To our knowledge, no prior study has employed BPM to examine a sample of patients with BPD.

The attitudinal body image differs from the perceptual body image (25) and the body schema (26). Attitudinal body image encompasses conceptual knowledge of the body at the declarative level and contains information about self-esteem and satisfaction related to one's body. We assessed this aspect through the body attitude test, a self-reported Likert scale. This test, renowned for its reliability and validity, has been extensively used among patients with eating disorders (27). However, its application within the borderline population is unprecedented.

The previous studies used self-report questionnaires or cognitive tasks to measure the internal image of one's body (10). Our research measured both body schema and perceptual body image on a somatosensory level, employing a tactile, vestibular, and visual input device. In this study, we have assumed that body schema and perceptual body image will show a moderately strong relationship with each other, while attitudinal body image will differ from these two types of representations. Previous studies have indicated disturbances in BPD patients' attitudes toward their bodies (6, 8), and we postulate that their perception of numerous somatosensory inputs could also be altered. Considering this, we hypothesized that attitudinal body image, body schema, and perceptual body image would be impaired in BPD patients compared to healthy controls. To our knowledge, our study is the first to examine the diverse forms of body representation in BPD through an experimental somatosensory approach.

## 2. Method

### 2.1. Procedures

Participants with BPD were recruited from the Department of Psychiatry and Psychotherapy inpatient unit at Semmelweis University, Budapest, Hungary. These patients took part in a 4-week psychotherapy program. The department's trained and experienced diagnosticians conducted diagnostic interviews before starting the therapy program. We applied availability sampling to recruit the healthy control group. The experimental and control groups were matched on age. Before the start of therapy, psychologists and physicians performed mental health screening with the participants using the Structured Clinical Interview for DSM-5-Personality Disorder [SCID-5-PD; (28)] and the Mini International Neuropsychiatric Interview [MINI; (29, 30)]. Neither the patients nor the controls received any payment for their participation in the study. Written informed consent was obtained from all participants and was approved by the Medical Research Council's National Scientific and Research Ethics Committee.

### 2.2. Participants

For the study, 31 patients were registered in the BPD group, along with 30 healthy subjects.

The inclusion criteria in the BPD group met the BPD diagnostic criteria of SCID-5-PD. In the case of healthy controls, inclusion criteria included a lack of current or previous psychiatric or neurological disease. A healthy participant who did not complete the questionnaires was excluded. The BPD group included six men (*19.4%*) and 25 women (*80.6%*). The mean age of the group was 28.38 years (*SD* = *8.853*). The youngest participant was 18 years old, and the oldest was 53 years old. The control group included 11 men (*36.7%*) and 19 (*63.3%*) women. The mean age was 28.93 years (*SD* = *8.489*). The youngest participant was 21, and the oldest was 54. Data relating to participants' education levels are presented in Table 1. A chi-square test was performed to examine the differences between BPD patients and healthy controls at the highest level of education. There was a significant difference between the groups: χ^2^ (4, *N* = 61) = 16.33, *p* = 0.03.

**Table 1:** Distribution of the highest educational level in the examined groups.

**Level of highest education**	**Frequency (percentage)**	
	**BPD group; *N* (%)**	**Healthy controls; *N* (%)**
Elementary school	5 (16.1)	0 (0)
Graduation in secondary high school	15 (48.4)	7 (23.3)
Collage	3 (9.7)	5 (16.7)
University	6 (19.4)	18 (60)
Postgraduate	2 (6.5)	0 (0)

Within the BPD group (*N* = *31*), 13 participants were diagnosed with one or more comorbid personality disorders at the time of the study [detected by the SCID-5-PD interview (*n* = *4* narcissistic, *n* = *2* avoidant, *n* = *2* obsessive-compulsive, *n* = *1* histrionic, *n* = *1* dependent, and *n* = *3* obsessive-compulsive and avoidant personality disorders)]. Current clinical disorders (formerly “Axis I” disorders) were diagnosed using the MINI in the BPD group. The most common comorbid Axis I disorders were major depressive episodes (*n* = *15, 48.4%*), anxiety disorders (*12, 38.7%*), and alcohol and/or drug abuse (*n* = *7, 22.6%*). Only four patients (*12.9%*) met the criteria of eating disorders (bulimia or anorexia nervosa), and in the case of three *(9.7%)* patients, body dysmorphic disorder was diagnosed.

### 2.3. Materials

#### 2.3.1. Body attitude test

The body attitude test (BAT) is a self-report questionnaire developed specifically for patients suffering from eating disorders (27, 31). Participants' agreement with 20 statements is assessed using a 6-point Likert-type scale ranging from 0 = never to 6 = always. The BAT uses three factors to assess one's negative appreciation of body size: lack of familiarity with one's body and general body dissatisfaction. Its psychometric characteristics were also tested on patients and healthy controls (32). The Hungarian version of the original BAT (33) was used. The test demonstrated good reliability, with Cronbach's alpha values between *a* = *0.6* and *a* = *0.849* for all scales in the BPD group and between *a* = *0.751* and *a* = *0.851* for all scales in healthy controls. We used the factors of the BAT as variables of attitudinal body image.

#### 2.3.2. Body portraying method

Body representation was assessed using a tool (the body portraying method) created by Verseghi and Nagy (24). At the Open Science Foundation (https://osf.io/7afd5/), the guide and the syntax of the PicMea program are freely available. The idea of the BPM is that the body's shape can be represented by a few typical spots (top of the head, neck, shoulder, armpit, waist, and several spots along the spine). Thus, we can observe how patients portray their bodies using these body areas (18, 24). Using this method, we touch these points on a participant's body and ask them to show where they felt the touch on a piece of paper (portraying a picture of the outline of a human body) in front of them, such as one would do in a mirror, and we mark them on that paper as well (for more details, see *Procedures section*). After that, we measured and marked the points that were touched on the participant's body. We then measured the difference between the points felt by the participants and the points touched in real life and used the sum of these as variables. We repeated the test with and without blindfolding the participants. Altogether, two variables were used: in the blindfolded condition, we measured body schema, and in the open-eyed condition, we measured perceptual body image.

##### 2.3.2.1. Procedure for body portraying method

Participants filled out the body attitude test in person before the BPM was recorded.

**Figure 1:**
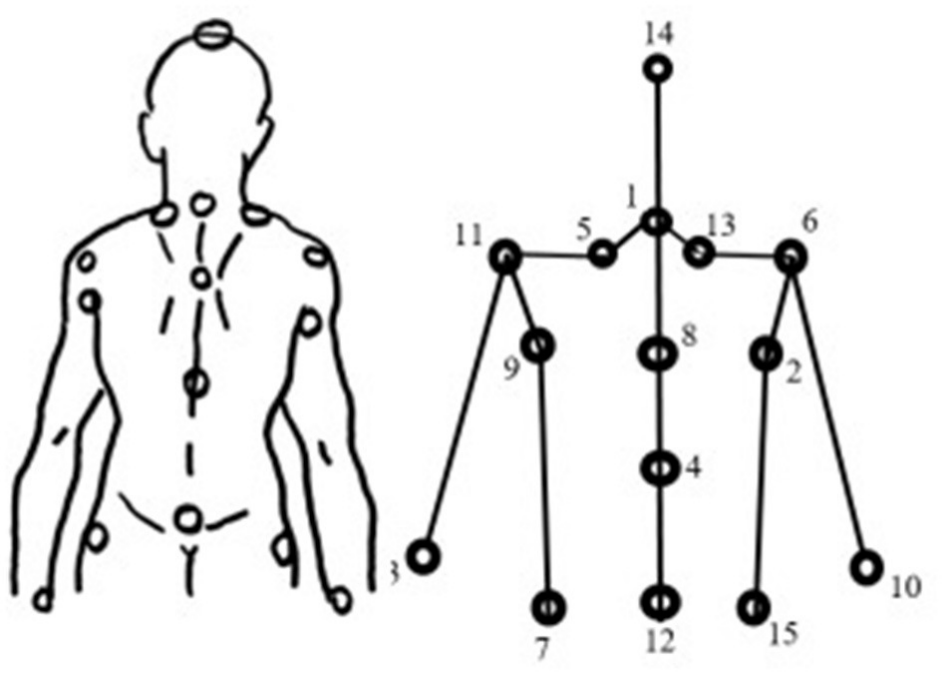
Schematic portrayal of the body using typical points. Numbers next to the body spots represent the sequence of the portrayal.

The BPM started with the instructions: “Imagine that this paper on the wall is a mirror! (At the same time, we pointed to a large piece of paper hanging on the wall. I will ask you to stand in front of this mirror and not move. I will touch a few spots on your body. As if you were seeing yourself in that mirror, please point out where you felt those touches on the paper. We will repeat this task twice: first blindfolded, then without a blindfold”. The participants were asked to close their eyes and were touched in strict order on various body parts by an experimenter standing behind them (Figure 1). The participants were asked to point to the area on the paper where they felt the touch. The experimenter marked these spots and gave them a predetermined sign. At the end of the task, participants were asked not to move from their position while the actual touch positions were recorded (the original points projected perpendicularly were marked on the paper). The procedure was then repeated without the blindfold.

**Figure 2:**
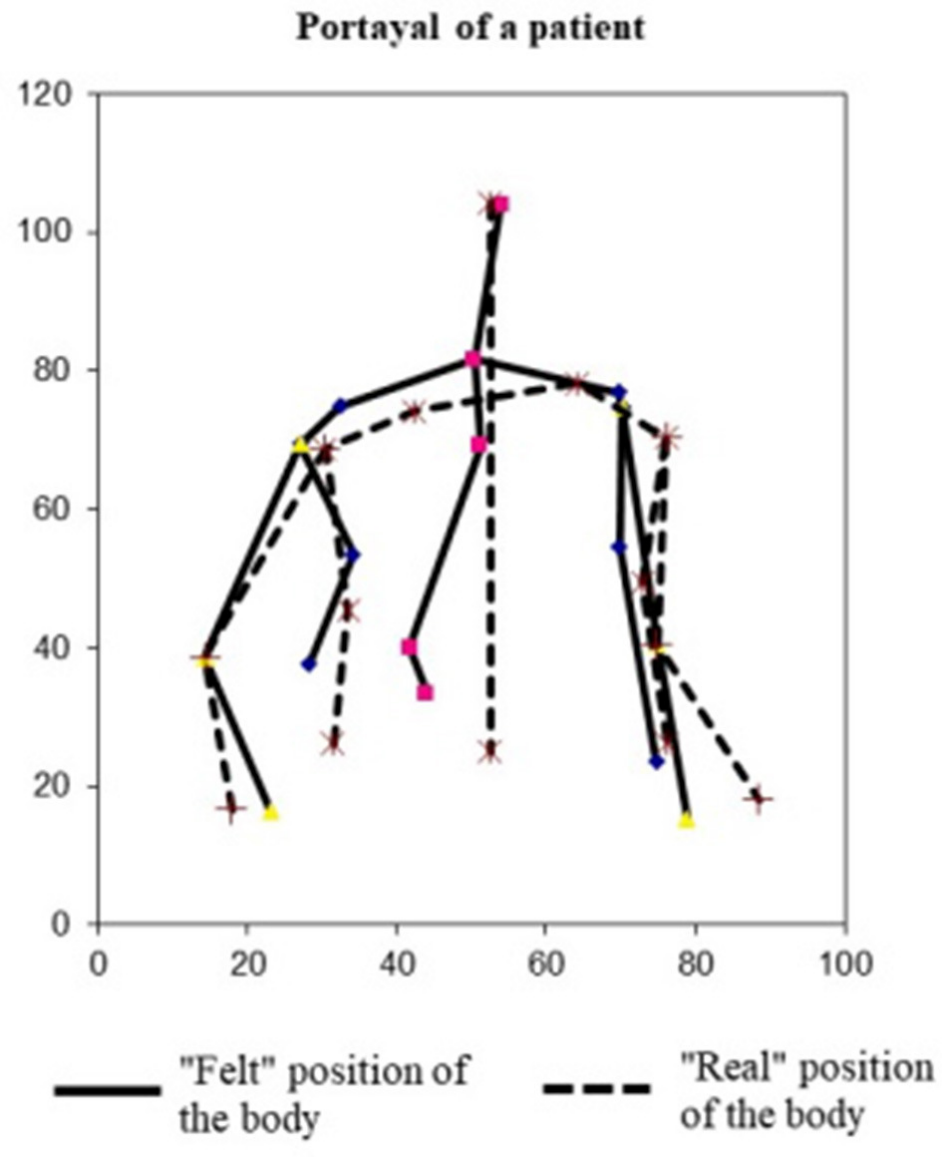
Example of a body portrayal of a patient subject. Solid lines show the body portrayal created by the subject; broken lines show the real position and shape of the subject's body.

A photo of each body-touch portrayal was taken and loaded into the PicMea program (a program made by Tibor Varga), which was able to register the coordinates of each point of the portrayals. The PicMea program is freely available at https://osf.io/7afd5/. On each photo, we clicked on each point (first the felt and second the real point) in the following order (see Figure 1): 1. Head (Nr. 14), 2. Points of the spine (Nr. 1, 8, 4, 12, up to down), 3. Left waist (Nr. 7), 4. Left armpit (Nr. 9), 5. Left shoulder (Nr. 11), 6. Left neck (Nr. 5), 7. Right neck (Nr. 13), 8. Right shoulder (Nr. 6), 9. Right armpit (Nr. 2), 10. Right waist (Nr. 15), 11. Points of elbows, 12. Left wrist (Nr. 3), and 13. Right wrist (Nr. 10). The program measures elbow spots (which were not part of this protocol), for which we chose random points that were later removed from the database. The real position of the spine was automatically calculated from the head point by the program. Therefore, we did not register that manually. After all points were registered to the program, we copied the coordinates into Excel, where we calculated the average of the differences between the observed and real points in the open and closed eye conditions using different formulas. In Excel, we could create the portrayal of the participants (see Figure 2).

### 2.4. Data analysis

All calculations were performed using IBM SPSS Statistics 26.0 (34).

Due to the small sample size, we used an independent *t*-test with bias-corrected and accelerated (BCa) bootstrapping (using 1,000 bootstrap samples, 95% confidence interval, CI) to examine the attitudinal body image, body schema, and perceptual body image differences (first, second, and third hypotheses) between the BPD group and the healthy control. We used an online calculator to examine the effect size.^1^ Because of limited resources, we could collect data from only 61 subjects. We applied sensitivity power analysis for the independent *t*-test using G^*^Power 3.1.9.4 (35) to calculate the minimum detectable effect size with α=0.05, 80% power, and the two groups' sample sizes were 31 and 30, respectively. The minimum detectable effect size was *d* = 0.64. We also reported the power of our significant results with smaller effect sizes.

To examine the interactions between attitudinal body image, perceptual body image, and body schema (fourth hypothesis), we used Pearson correlation and considered a correlation coefficient (r) of 0.1 as a small, 0.3 as a medium, and 0.5 as a large effect size.

Differences between the BPD group's and healthy controls' correlation were calculated each time with Fischer's z-test, for which we used an online calculator as well.^2^ A *p* ≤ 0.05 was considered significant.

There was no missing data in the case of BAT. In the case of BPM, in four cases, we were not able to register data for a single pot due to some physical discrepancy in the body. These points were considered zeros. In one case, for example, the participant's hand was tied up, so we marked the position of his wrist at the same point in the program in both the felt and the real case. This created a zero value in the pot.

The Regional and Institutional Committee of Science and Research Ethics of Semmelweis University approved the research procedure (Nr. 80/2019).

## 3. Results

### 3.1. Attitudinal body image in BPD patients and healthy controls

First, we examined the differences in factors of the body attitude test between the BPD group and healthy controls with independent *t*-tests with bias-corrected and accelerated bootstrapping (see Table 2). The analysis indicated significant differences between the examined groups in the case that all factors have a larger effect size than the minimum detectable effect size *d* = 0.64, as indicated by our sensitivity power analyses.

**Table 2:** Comparison of the BPD and the healthy group regarding the factors from the body attitude test and the measures from the body portraying method.

**Measures**	**M (SD)**		***t*-test (df = 59)**	**Bootstrap (95% CI)**				
				**d (*p*)**	**Lower**		**Upper**	
	**BPD group**	**Healthy controls**			**BPD group**	**Healthy controls**	**BPD group**	**Healthy controls**
Lack of familiarity with one's own body	17.81 (5.07)	8.53 (5.81)	6.65	1.7 (0.001)	15.9	6.41	19.69	10.71
Negative appreciation of body size	18.67 (9.2)	8.23 (7.05)	4.97	1.27 (0.001)	15.29	5.55	22.12	10.84
General dissatisfaction	13.36 (4.78)	7.43 (4.8)	4.83	1.24 (0.001)	11.45	5.68	15.09	9.29
Body schema	10.90 (3.65)	9.10 (2.7)	2.19	0.56 (0.03)	9.69	8.24	12.16	9.93
Perceptual body image	10.72 (3.44)	8.44 (2.76)	2.84	0.75 (0.01)	9.68	7.57	11.79	9.34

d = Cohen'd, CI = 95% bootstrap confidence interval.

### 3.2. Perceptual body image and body schema in BPD patients and healthy controls

The differences in variables of the BPM (which measured body schema and perceptual body image) were examined with an independent *t*-test with bias-corrected and accelerated bootstrapping (see Table 2). The analysis indicated significant differences between the BPD group and healthy controls in the cases of perceptual body image with a large effect size (*d* = 0.75) and body schema with a medium effect size (*d* = 0.56). The latter effect size is smaller than our sensitivity power analyses' minimum detectable effect size (*d* = 0.64). In the case of our sample size, the power to detect *d* = 0.56 effect size is 70%, so it needs to be interpreted cautiously.

To ensure that the group differences were not driven solely by comorbid eating disorders or body dysmorphic disorders, we ran additional calculations. We excluded individuals with these conditions from the BPD sample before reanalyzing the comparison between BPD and healthy subjects. Similar to the first calculation, we found significant differences between the experimental and control groups in the cases of body schema {t_(55)_ = −2.39, *p* = 0.026; 95% CI [−3.81, −0.37]}, perceptual body image {t_(55)_ = −2.75, *p* = 0.008; 95% CI [−3.77, −0.69]}, lack of familiarity with one's own body {t_(55)_ = −6.31, p = 0.001; 95% CI [−12.35, −6.57]}, negative apperception of body size {t_(55)_ = −4.46, p = 0.001; 95% CI [−14.44, −5.47]}, and general dissatisfaction {t_(55)_ = −4.25, p = 0.001; 95% CI [−7.82, −2.82]} as well.

### 3.3. Relationship between body schema, perceptual body image, and attitudinal body image

The relationship between body schema and perceptual body image was calculated. We found a strong association between these variables in the case of the BPD group (*r* = 0.81; *p* < 0.001) and the healthy controls as well *(r* = 0.66*; p* < *0.001)*. There was no significant difference between the magnitudes of the correlations of the examined groups *(z* = *1.25; p* = 0.212*)*.

A comparison of factors that measured attitudinal body image (lack of familiarity with one's own body, negative appreciation of body size, and general dissatisfaction) and the body schema did not show a significant relationship, either in the case of BPD or in the case of healthy controls (see Table 3). The same non-significant results in comparing factors of attitudinal body image and perceptual body image were reached. There was no significant difference between the magnitudes of the correlations of the examined groups (*all ps* > 0.05*;* see Table 3).

**Table 3:** Associations between factors of the body attitude test and variables of body portraying method task.

**Measures**	**Groups**	**Lack of familiarity with one's own body**	**Negative appreciation of body size**	**General dissatisfaction**
Body schema	BPD group/healthy control	0.27/0.16	0.16/0.17	0.32/0.2
	z (*p*)	0.43 (0.67)	−0.04 (0.45)	0.48 (0.63)
Perceptual body image	BPD group/healthy control	0.06/0.12	−0.94/0.18	0.2/0.24
	z (*p*)	−0.22 (0.82)	−1.02 (0.31)	0.16 (0.43)

z = value of Fischer's z test.

## 4. Discussion

### 4.1. Body schema and perceptual body image in BPD

According to our first and second hypotheses, we anticipated less accurate body schema and perceptual body image in borderline personality disorder compared to healthy controls. Patients with BPD had higher scores on these scales, meaning they had significant disturbances in body schema and perceptual body image. The findings of our study provide the first objective and experimental evidence that strengthens the idea of a disrupted internal model of the body in BPD, not only on perceptual but also somatosensory levels of body representation.

These results align with previous studies, which found reduced whole-body ownership (8), altered touch perception (6), enhanced body plasticity (36), and higher susceptibility to the rubber hand illusion (37), all of which point to perturbed body perception in BPD.

One conceivable interpretation of our findings may be linked to dissociative symptoms, such as depersonalization, frequently observed in BPD (38). Neuroimaging studies found that depersonalization is associated with functional abnormalities along sequential hierarchical areas of the sensory cortex (visual, auditory, and somatosensory) and areas responsible for an integrated body schema (39). Our results indicate that BPD's functionally compromised somatosensory system could potentially contribute to dissociative symptoms. However, this hypothesis necessitates further exploration.

The somatosensory system remains in constant development, particularly during early childhood, and adapts to changing circumstances (18, 40). It has demonstrated the ability to reorganize the somatosensory cortex following limb amputations (40) and instances of sexual abuse (41). In subsequent research, it would be pertinent to investigate whether a higher incidence of early childhood traumas and adverse life events correlates with elevated levels of body schema and perceptual body image distortions.

### 4.2. Body attitude in borderline personality disorder

According to our third hypothesis, we expected a disrupted attitudinal body image in BPD patients.

Borderline patients scored higher on all factors of the BAT than healthy controls. The results of BAT suggest that those with BPD perceive certain body parts as excessively large or fat, experience heightened anxiety and distress regarding their bodies, and express greater dissatisfaction with their physical appearance than their healthy counterparts. These results tie well with previous studies, wherein it has been found that patients with BPD have negative attitudes toward their body more often (10, 11) and show high levels of dissatisfaction with their body parts that are as high as those found in patients with bulimia nervosa (10, 42). One of the studied subscales in the BPD population showed a strong correlation with the frequency of BPD symptoms (12), which is also consistent with our findings. Our results also follow the study of Sansone, Wiederman, and Monteith (4), where borderline personality disorder symptom scores were negatively associated with self-rated bodily and facial attractiveness.

### 4.3. Comparison of body schema, perceptual body image, and attitudinal body image

According to our fourth hypothesis, we expected a stronger relationship between body schema and perceptual body images than between these variables and attitudinal body image.

The internal model of our body is composed of different representations based on different somatosensory and cognitive-processing levels of body representation systems (18). This system includes body schema, perceptual body image, and attitudinal body image. While body schema and perceptual body image are related to the tactile-kinaesthetic and/or visual sensory systems, attitudinal body image was conceptualized on a verbal-symbolic level. Consequently, attitudinal body image diverges more markedly from the other two measured representations, elucidating why body schema and perceptual body image exhibit a more robust interrelationship than attitudinal body image. This result fits well with previous neuropsychological studies on neglect syndrome (24, 43) or brain injury (44), suggesting that damage in body schema or perception of one's body can be observed without altering attitudes toward the body. Another study confirmed this finding, revealing a clear difference between attitudinal and perceptual body image (25). By summarizing this, we identified new layers of body representations, body schema, and perceptual body image that are relatively independent of the previously studied attitudinal body images.

### 4.4. Conclusion

Our study's objective was to investigate, besides cognitive-level, attitude-based body image, the deeper somatosensory and visually-based body representations in individuals with borderline personality disorder. Our findings revealed disrupted body representations at all examined levels in patients with BPD compared to the healthy control group. Our results suggest that individuals with BPD exhibit disturbances not only in terms of negative cognitions and emotional responses toward their bodies but also in terms of visual and tactile-kinesthetic perceptions of bodily experiences. The therapeutic interventions for BPD aim to change patients' negative thoughts, beliefs, and schemas, and these treatments may affect attitude-based body image (19, 39). However, very little is known about therapies focusing on the body schema in BPD. New therapeutic interventions for modifying body image, such as virtual reality and multisensory feedback interventions, which are promising to treat distorted body images (45), multisensory spatial interactions for stimulation on or around our body parts, which may affect our spatial perception of touch and the disposition of our body (20), and dance movement therapy (40), may be the target of new psychotherapy intervention research in the treatment of BPD.

Based on our results, it is suggested that more complex, body-focused, and movement-centered supplementary therapeutic interventions may be necessary for the treatment of BPD.

Further research will be aimed at exploring the underlying factors behind the results. To the best of our knowledge, this is the first occasion where different levels of body representation were examined in one study.

### 4.5. Limitations

Because of our limited resources, our sample size was lower than optimal. We did not collect refusal rate data, so our sample may be biased in a systematic way. Our study highlights a particularly important difference between patients and healthy people; however, we are not able to explore the causal roots of body-image disturbances. Future research must be crucial to clarifying the causal factors behind the differences between the examined groups.

## Data availability statement

The original contributions presented in the study are included in the article/supplementary material, further inquiries can be directed to the corresponding author.

## Ethics statement

The studies involving humans were approved by the Regional and Institutional Committee of Science and Research Ethics of Semmelweis University approved the research procedure (Nr. 80/2019). The studies were conducted in accordance with the local legislation and institutional requirements. The participants provided their written informed consent to participate in this study.

## Author contributions

ZU, ZN, and DSzi contributed to conception and design of the study. DSzi and RK organized the database. DSza performed the statistical analysis. DSzi wrote the first draft of the manuscript. ZU, ZN, DSzi, DSza, and RK wrote sections of the manuscript. All authors contributed to manuscript revision, read, and approved the submitted version.

## Footnotes

^1^ https://www.socscistatistics.com/effectsize/default3.aspx

^2^ http://vassarstats.net/rdiff.html?
